# PGI_2_ signaling metabolically reprograms CD4 Th2 cells and represses allergic airway inflammation

**DOI:** 10.1093/jimmun/vkaf130

**Published:** 2025-06-30

**Authors:** Weisong Zhou, Jian Zhang, Nowrin U Chowdhury, Allison E Norlander, Shinji Toki, Masako Abney, Mark Rusznak, Katherine N Gibson-Corley, Daniel P Cook, Dawn C Newcomb, Ray Stokes Peebles

**Affiliations:** Division of Allergy, Pulmonary and Critical Care Medicine, Department of Medicine, Vanderbilt University School of Medicine, Nashville, TN, United States; Division of Allergy, Pulmonary and Critical Care Medicine, Department of Medicine, Vanderbilt University School of Medicine, Nashville, TN, United States; Children’s Hospital of Philadelphia, Philadelphia, PA, United States; Department of Anatomy, Cell Biology and Physiology, Indiana University School of Medicine, Indianapolis, IN, United States; Division of Allergy, Pulmonary and Critical Care Medicine, Department of Medicine, Vanderbilt University School of Medicine, Nashville, TN, United States; Division of Allergy, Pulmonary and Critical Care Medicine, Department of Medicine, Vanderbilt University School of Medicine, Nashville, TN, United States; Division of Allergy, Pulmonary and Critical Care Medicine, Department of Medicine, Vanderbilt University School of Medicine, Nashville, TN, United States; Division of Comparative Medicine, Vanderbilt University School of Medicine, Nashville, TN, United States; Department of Pathology, Microbiology, and Immunology, Vanderbilt University School of Medicine, Nashville, TN, United States; Department of Internal Medicine, University of Iowa, Iowa City, IA, United States; Division of Allergy, Pulmonary and Critical Care Medicine, Department of Medicine, Vanderbilt University School of Medicine, Nashville, TN, United States; Division of Allergy, Pulmonary and Critical Care Medicine, Department of Medicine, Vanderbilt University School of Medicine, Nashville, TN, United States; Department of Pathology, Microbiology, and Immunology, Vanderbilt University School of Medicine, Nashville, TN, United States; Veterans Affairs Tennessee Valley Health Care, Nashville, TN, United States

**Keywords:** inflammation, lung, mouse, T cells

## Abstract

Prostaglandin I_2_ (PGI_2_) is a lipid mediator known to inhibit T helper 2 (Th2) immune responses and allergic inflammation, but its role in regulating Th2 cell metabolism remains underexplored. Using the Seahorse assay, we evaluated the effects of PGI_2_ signaling on Th2 cell glycolysis and mitochondrial respiration. Our results show that cicaprost, a stable PGI_2_ analog, significantly reduced basal, maximal, and spare glycolytic capacities in wild-type Th2 cells, while these effects are absent in Th2 cells lacking the PGI_2_ IP receptor (IP knockout [KO]). Cicaprost also impaired mitochondrial respiration and adenosine triphosphate production in wild-type Th2 cells, but not in IP KO cells, indicating that PGI_2_ signaling is essential for these metabolic changes. Further analysis revealed that cicaprost decreased glucose transporter 1 expression and glucose uptake and inhibited mitochondrial mass and membrane potential, suggesting that PGI_2_ signaling inhibits Th2 cell metabolism by reducing glucose availability and mitochondrial respiratory functions. Metabolomic profiling of cicaprost-treated Th2 cells showed dose-dependent changes, with 126 downregulated and 233 upregulated metabolites showing over 2-fold significant changes compared with vehicle-treated cells. Pathway analysis of these altered metabolites suggests a shift from catabolism to anabolism in cicaprost-treated Th2 cells. In vivo, CD4-specific conditional IP KO mice (CD4^Cre+^IP^flox^) exhibited exacerbated lung inflammation following exposure to *Alternaria alternata* extract, marked by increased IL-5 and IL-13 production, enhanced eosinophilia, and elevated mucus production. These findings establish a mechanistic link between PGI_2_-mediated immunoregulation and metabolic reprogramming, reinforcing its role as a key modulator of allergic inflammation.

## Teaser

The Editors have selected this article as a highlight of the issue.

## Introduction

The differentiation and effector functions of CD4 T helper 2 (Th2) cells drive the pathogenesis of allergic diseases like asthma. Th2 cells are distinguished by their production of cytokines such as interleukin (IL)-4, IL-5, and IL-13, which mediate processes such as IgE class switching, eosinophil recruitment, and mucus production. Understanding the regulation of Th2 cell metabolism and its impact on their function is an emerging area of interest with substantial therapeutic potential.^1^

Metabolic reprogramming is a hallmark of T cell activation and differentiation. Upon activation, T cells increase glucose uptake and rely on glycolysis to meet their energy and biosynthetic demands, even in the presence of oxygen.^2^ Mitochondrial respiration also contributes to adenosine triphosphate (ATP) production and supports various biosynthetic processes essential for T cell function.^3^^,^^4^ Dysregulation of these metabolic pathways can significantly impact T cell function and the overall immune response.^5^^,^^6^ Metabolic pathways such as glycolysis and oxidative phosphorylation are essential for Th2 cell polarization and function.^1^ Recent studies have revealed that different T cell subsets utilize distinct metabolic programs to support their specialized functions. While both Th2 and Th17 cells drive allergic airway inflammation through distinct metabolic programs—with Th17 cells relying on glutaminolysis and showing sex-specific androgen regulation^7^ and Th2 cells exhibiting elevated glucose metabolism^8^—the upstream mediators controlling these pathways remain poorly understood.

Prostaglandin I_2_ (PGI_2_), a lipid mediator in the cyclooxygenase metabolic pathway, has been shown to suppress Th2 immune responses and allergic lung inflammation through interaction with the IP receptor.^9–12^ However, its specific effects on Th2 cell metabolism remains unclear. We hypothesize that PGI_2_ signaling suppresses Th2 cell metabolism, including glycolysis and mitochondrial respiration. Understanding this relationship could reveal new strategies to modulate Th2 cell function in allergic diseases.^6^

To investigate this, we used wild-type (WT) and IP receptor knockout (IP KO) mice. Additionally, we generated CD4^Cre+^IP^flox^ mice, a genetic model with specific deletion of the IP receptor in CD4^+^ cells, to investigate the effects of PGI_2_ on Th2 cells in the context of allergic lung inflammation. We hypothesized that CD4^Cre+^IP^flox^ mice would exhibit enhanced Th2-mediated allergic inflammation compared with CD4^Cre-^IP^flox^ mice, highlighting the critical role of IP signaling in Th2 cell responses. By integrating metabolic assays with genetically modified mouse models, this study provides a comprehensive mechanism through which PGI_2_ signaling downregulates Th2 cell–mediated immune responses. These findings could help identify new metabolic checkpoints for treating Th2-mediated allergic diseases.

## Materials and methods

### Mice

IP-deficient (IP KO) mice on a C57BL/6 genetic background were provided by Dr Garret Fitzgerald (University of Pennsylvania) and backcrossed to BALB/c for more than 10 generations in our lab. Wilt type (WT) BALB/c mice and CD4^Cre^ mice on a C57BL/6 background were purchased from the Jackson Laboratory.

*Ptgir ^fl/fl^* (IP^flox^) mice were generated using split-GFP technology, developed by Ingenious Targeting Laboratory. These mice were designed such that Cre-mediated deletion of the targeted gene activates a reporter gene, such as GFP or another fluorescent marker, to label the cells where the gene has been knocked out. Specifically, the IP gene (*Ptgir*) was identified, and critical exons were flanked with loxP sites within the targeting vector. This vector also included a neomycin resistance gene flanked by flippase (FLP) recombination target (FRT) sites for positive selection and a cerulean fluorescent reporter gene, which was split into two parts. The 5′ portion of the cerulean gene was positioned at the end of the IP gene, while the 3′ portion was placed in intron 1. Upon Cre recombination, these two parts of the cerulean gene were spliced together, resulting in functional cerulean fluorescence in cells where the IP gene was deleted.

The targeting vector was electroporated into FLP C57BL/6 mouse embryonic stem (ES) cells, which then selected using the G418 antibiotic. Correctly targeted ES cells were confirmed through polymerase chain reaction (PCR), real-time polymerase chain reaction (RT-PCR), and sequencing analyses. The neomycin resistance gene was removed by transient expression of FLP recombinase. These validated targeted ES cells were injected into BALB/c blastocysts, which were subsequently implanted into pseudopregnant female mice to generate chimeric mice. The chimeric mice were bred with C57BL/6 WT mice to produce heterozygous floxed offspring, verified by PCR. Heterozygous floxed mice were interbred to obtain homozygous floxed offspring (IP^flox^), confirmed by genotyping. The validation process included verifying correct loxP insertion and ensuring intact gene function until Cre-mediated recombination. IP^flox^ mice and CD4^cre^ mice were used to generate CD4^Cre-^IP^flox^ and CD4^Cre+^IP^flox^ mice through multiple rounds of breeding. Functional assays with Cre-expressing mice confirmed the successful generation of the conditional KO with the cerulean reporter.

Age-matched female mice (8–12 wk old) were used for all experiments. All animal procedures were reviewed and approved by the Institutional Animal Care and Use Committee at Vanderbilt University.

### Purification and culture of CD4 T cells

Naive CD4 T cells of WT and IP KO mice were purified from mouse spleens using the STEMCELL Technologies Naive CD4 T Cell Isolation Kit. After preparation of single-cell suspensions, non-CD4 cells were depleted using streptavidin-coated magnetic particles, leaving untouched naive CD4 T cells in the supernatant. Purified CD4 T cells were resuspended in complete RPMI 1640 medium supplemented with 10% fetal bovine serum, 4 mM L-glutamine, 1 mM sodium pyruvate, 55 µM beta-mercaptoethanol, 10 mM HEPES, and 100 units/mL penicillin and 100 µg/mL streptomycin. T cells were activated with plate-bound anti-CD3 and anti-CD28 (1 µg/mL each in phosphate-buffered saline [PBS]) in flat-bottom non–tissue culture–treated 24-well plates (Corning; reference number 351172). The cells were cultured under Th2 polarization conditions with mouse IL-4 (10 ng/mL) and anti-mouse IFN-γ (10 µg/mL), and treated with the stable PGI_2_ analog, cicaprost, at concentrations of 2 nM, 20 nM, 200 nM, or vehicle (DI water) as control. The treatment lasted for 3 d for Th2 cytokine production, glucose transporter 1 (GLUT1) and arginase 1 (Arg1) expression, glucose uptake, and mitochondrial biogenesis and function assays, or 6 d for Seahorse Glyco Stress, Mito Stress, and ATP production rate assays.

### Seahorse assay

Th2 cell metabolism was evaluated using the Agilent Seahorse Extracellular Flux Analyzer (Agilent Technologies). Cells were seeded in Seahorse XF Cell Culture Plates at densities of 100,000 cells per well and incubated for 35 min at 37 °C in a non-CO_2_ incubator. The Seahorse XF Analyzer was calibrated using standard solutions and programmed to inject metabolic modulators: glucose, oligomycin, and 2-deoxy-D-glucose for Glyco Stress (glycolysis) assays, and oligomycin, FCCP, and rotenone/antimycin A for Mito Stress (mitochondrial respiration) assays. For the Real-Time ATP Production Rate Assay, injections of oligomycin and rotenone/antimycin A were used. Extracellular acidification rate and oxygen consumption rate were measured before and after each injection. Data were analyzed with Seahorse XF software, normalized to live cell counts.

### Glucose uptake assay

Glucose uptake in CD4 T cells of WT and IP KO mice, polarized to Th2 cells for 3 d, was measured using the Promega Glucose Uptake-Glo Assay Kit (Cat. J1342). Cells were treated with cicaprost at concentrations of 2 nM, 20 nM, and 200 nM before glucose uptake measurements.

### Metabolomics analyses for Th2 cells

Th2 cells activated and treated with cicaprost for 3 d were harvested for metabolomics analysis by the Center for Innovative Technology at Vanderbilt University. Metabolites were extracted from cell lysate using an MeOH:H_2_O (80:20) protein precipitation protocol, with normalization based on equal protein amount (40 µg per sample). The extracted metabolites were analyzed using hydrophilic interactive liquid chromatography in negative ion mode coupled to a Thermo Fisher Scientific Q Exactive HF (LC-Hybrid Quadrupole-Orbitrap MS/MS) mass spectrometer. The resulting data were processed and aligned with reference metabolite databases for identification and quantification using Progenesis QI 2.0 and MetaboAnalyst 4.0.

### Real-time PCR

To confirm the deletion of IP in CD4 T cells, RNA was extracted from purified CD4 T cells of CD4^Cre-^IP^flox^ and CD4^Cre+^IP^flox^ mice. Complementary DNA synthesis and RT-PCR were performed, followed by agarose gel electrophoresis to visualize the IP-specific product. Primers used for RT-PCR are 5′CCGCCAACAGAGACGCCACCAT-3′ (forward primer) and 5′CGGGCACACAGGCAACACAACCA-3′ (reverse primer). To quantify prostaglandin receptor expression via SYBR Green-based RT-PCR, the following Qiagen QuantiTect Primer Assays were used: PTGDR1 (DP1, catalog # QT00114310) and PTGDR2 (DP2/CRTH2, QT00320537) for PGD2 receptors; PTGER1 (EP1, QT00173936), PTGER2 (EP2, QT0000115276), PTGER3 (EP3, QT00254303), and PTGER4 (EP4, QT02589440) for PGE2 receptors; PTGFR (FP, QT00170562) for PGF_2_α receptor; PTGIR (IP, QT00160062) for the PGI_2_ receptor; and TATA box binding protein (Tbp, QT00198443). Slc7a2/Cat2b gene expression assay primers were purchased from Thermo Fisher Scientific (Mm01260610_m1).

### Induction of allergic lung inflammation

CD4^Cre-^IP^flox^ and CD4^Cre+^IP^flox^ mice were sensitized intranasally with *Alternaria alternata* extract (Alt) (Greer Laboratories; 2 µg in 40 µL PBS per mouse) or PBS as a control for 3 d. After a 13-d rest period, mice were challenged with Alt (0.125 µg in 40 µL PBS per mouse) for 2 consecutive days. On day 17, bronchoalveolar lavage fluid (BALF) and lung tissue were collected for analysis of inflammation and cytokine levels.

### BALF collection and differential cell count

BALF was collected by instilling 800 µL of PBS into the lungs via tracheostomy, followed by gentle suction. Total white blood cells were counted, and cytospin slides were prepared for differential cell counts to identify eosinophils, lymphocytes, neutrophils, and macrophages.

### Histopathology of lungs

Lungs were inflated with PBS, harvested, and fixed in 10% neutral buffered formalin, then processed and embedded in paraffin blocks. Tissue sections were stained with periodic acid-Schiff (PAS) to assess mucus production or with hematoxylin and eosin to evaluate inflammatory cell infiltration. Slides were examined and scored by a board-certified veterinary pathologist who was blinded to the groups. Airway mucus in epithelial cells of bronchioles was scored on a scale from 0 to 3: no PAS staining (score 0), very rare PAS positivity in major/large airways (score 1), scattered PAS positivity in large and medium sized airways (score 2), or multifocal to diffuse PAS positivity in many airways regardless of size (score 3). Perivascular inflammation and peribronchiolar inflammation were scored on a scale from 0 to 3: not present (score 0); mild, rare, scattered inflammation (score 1); moderate, multifocal inflammation (score 2); and marked, locally extensive to diffuse inflammation (score 3).

### Flow cytometry

Th2 cells treated with vehicle or cicaprost for 3 d were surface stained with antibodies specific for CD3, CD4, and GLUT1. For intracellular protein staining, the cells were fixed, permeabilized, and stained with antibodies for GLUT1, Arg1, or IL-4. For dead cell exclusion, DAPI was used for surface staining and LIVE/DEAD Fixable Red Dead Cell Stain Kit (Thermo Fisher Scientific) for intracellular staining. For IL-4 intracellular staining, the cells were stimulated with PMA (50 ng/mL) and ionomycin (1 µg/mL) for 5 h prior to the flow cytometry staining protocol. MitoTracker Green FM (Thermo Fisher Scientific; Cat. M7514) and MitoSOX Red Mitochondrial Superoxide Indicator (Thermo Fisher Scientific; Cat. M36008) were used for flow cytometry staining to determine mitochondrial mass, membrane potential, and superoxide levels. Samples were analyzed using a BD LSR II or Cytek Aurora flow cytometer and data were processed with FlowJo v10 software (BD Biosciences).

### Enzyme-linked immunosorbent assay

Cytokines (IL-5, IL-13, and CCL11) were measured in lung tissue and Th2 cell culture supernatant using Quantikine ELISA kits (R&D Systems). The expression of mitochondrial proteins cytochrome c oxidase subunit 1 (COX-1) and succinate dehydrogenase subunit A (SDH-A) were analyzed using MitoBiogenesis In-Cell ELISA Kit (Abcam; Cat. ab110217) according to the manufacturer’s instructions.

### Statistical analysis

GraphPad Prism v10.4.2 software (GraphPad Software) was used for statistical analysis. Data were compared using 2-way analysis of variance with Sidak’s multiple comparison test for comparisons involving more than 2 groups. Results are presented as mean ± SEM, with *P* < 0.05 considered significant.

## Results

### PGI_2_ signaling suppresses Th2 cell glycolysis and mitochondrial respiration

**Figure 1.**
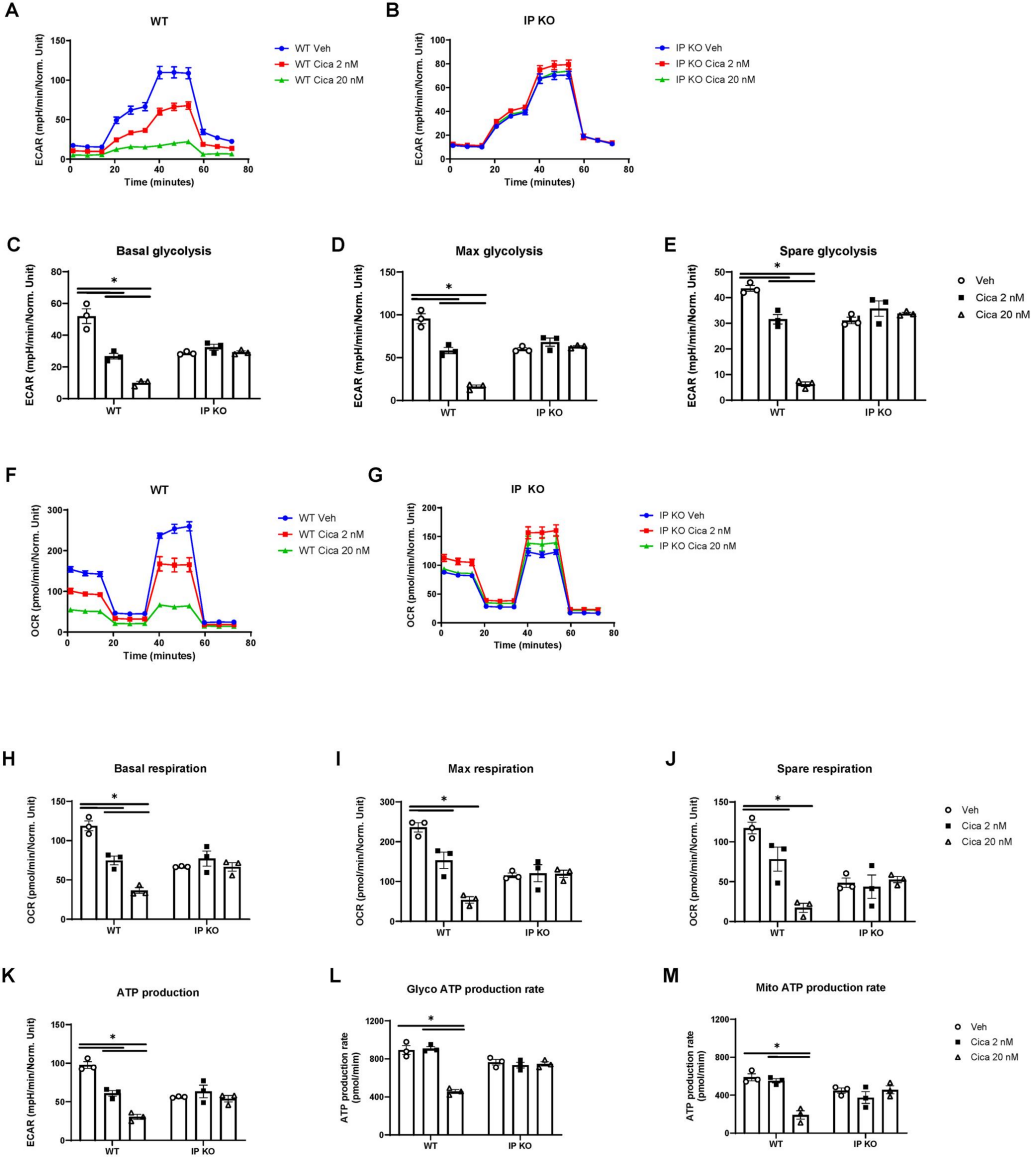
PGI_2_ signaling suppresses metabolic activity in Th2-polarized CD4^+^ T cells. Naïve splenic CD4^+^ T cells from WT and IP KO mice were activated with anti-CD3/CD28 under Th2-polarizing conditions (IL-4 + anti-IFNγ) and treated with vehicle (water) or cicaprost (2 nM or 20 nM) for 6 d. Metabolic profiles were assessed using Seahorse XF assays normalized to live cell counts. (A, B) Extracellular acidification rate (ECAR) (glycolysis). (C–E) Glycolytic parameters: basal, maximal, and spare glycolytic capacity. (F, G) Oxygen consumption rate (OCR) (mitochondrial respiration). (H–J) Mitochondrial parameters: basal, maximal, and spare respiratory capacity. (K) Total ATP production. (L) Glycolytic ATP production rate. (M) Mitochondrial ATP production rate. Data represent 3 (A–K) or 2 (L, M) independent experiments (n = 3 mice/group). *P < 0.05 by 2-way analysis of variance.

To investigate the role of PGI_2_ in Th2 cell metabolism, we assessed glycolysis and mitochondrial respiration using the Seahorse assay. Treatment with cicaprost, a stable PGI_2_ analog, significantly reduced basal, maximum, and spare glycolytic capacity in WT Th2 cells compared with vehicle-treated controls (Fig. 1A, C, E), while IP KO Th2 cells remained unaffected (Fig. 1B–E). This indicates that PGI_2_-IP signaling inhibits glycolysis, a critical pathway for cellular energy production and proliferation. The absence of this suppression in IP KO Th2 cells confirms that the effect is IP receptor dependent.

Similarly, cicaprost treatment impaired mitochondrial function in WT Th2 cells, reducing basal, maximum, and spare respiratory capacity (Fig. 1F, H–J) as well as ATP production and ATP production rate (Fig. 1K–M). These findings suggest that PGI_2_ disrupts mitochondrial oxidative metabolism and energy generation, with no observed effects in IP KO cells (Fig. 1G–M).

Consistent with these metabolic changes, reduced glycolysis and respiration correlated with diminished IL-5 and IL-13 production in WT but not in IP KO Th2 cells (Fig. S1A, B), aligning with our previous work.^13^ Furthermore, cicaprost decreased the total number of IL-4–producing WT Th2 cells and total live cell counts, with no impact on IP KO cells (Fig. S1C, F). Together, these results demonstrate that PGI_2_-IP signaling modulates Th2 cell function by suppressing metabolic pathways, thereby limiting cell expansion and cytokine production.

### PGI_2_ signaling inhibits Th2 cell expression and glucose uptake

**Figure 2.**
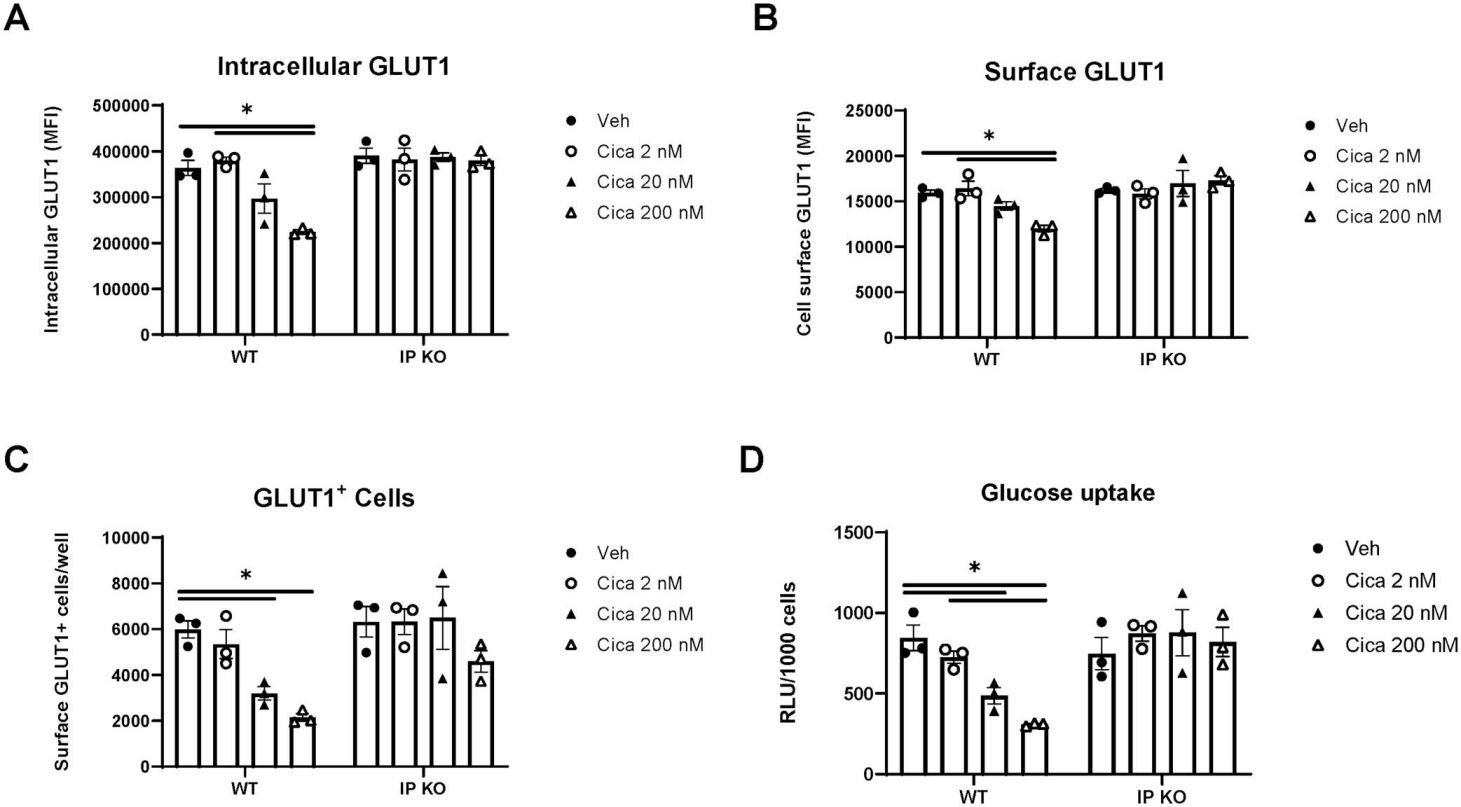
PGI_2_ signaling impairs glucose metabolism in Th2 cells through GLUT1 regulation. Naïve splenic CD4^+^ T cells from WT and IP KO mice were stimulated with anti-CD3/CD28 under Th2-polarizing conditions (IL-4 + anti-IFNγ) and treated with vehicle or cicaprost (2 nM, 20 nM and 200 nM) for 3 d. (A) Intracellular and (B) surface GLUT1 expression analyzed by flow cytometry (gated on live, single, CD3^+^CD4^+^, and GLUT1^+^ cells). (C) Total GLUT1^+^ CD4^+^ T cells. (D) Glucose uptake measured using Promega Glucose Uptake-Glo Assay (luminescence normalized to cell number, relative luminescence units [RLU]). Data represent 3 independent experiments (n = 3 mice/group). *P < 0.05 by 2-way analysis of variance. MFI, mean fluorescence intensity.

To investigate glycolytic suppression mechanisms, we examined GLUT1 expression and glucose uptake in Th2 cells. Cicaprost treatment reduced both total GLUT1 protein (Fig. 2A) and cell surface GLUT1 expression (Fig. 2B) specifically in WT Th2 cells, with no effect in IP KO cells. The population of surface GLUT1^+^ cells was similarly decreased in WT but not in IP KO T cells (Fig. 2C). These results indicate impaired GLUT1 biosynthesis and membrane translocation. Consistent with these observations, glucose uptake was diminished in WT but not in IP KO CD4^+^ T cells (Fig. 2D). These results demonstrate that PGI_2_-IP signaling restricts cellular glucose entry by downregulating GLUT1 expression and membrane localization, providing a molecular basis for the reduced glycolytic flux and metabolic activity in Th2 cells.

### PGI_2_ signaling inhibits Th2 cell mitochondrial mass and functions

**Figure 3.**
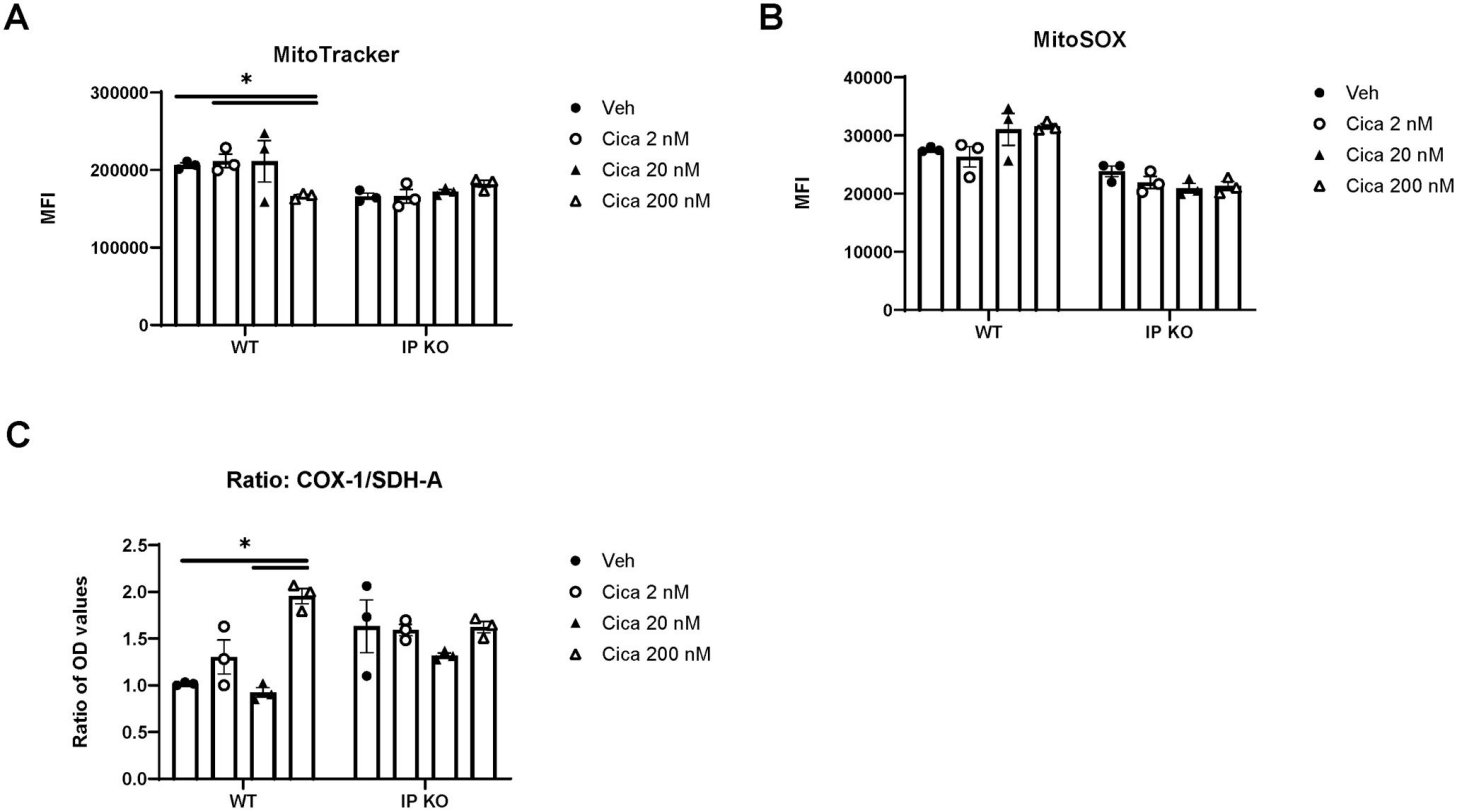
PGI_2_ signaling impairs mitochondrial integrity in Th2 cells. Naïve splenic CD4^+^ T cells from WT and IP KO mice were polarized under Th2-polarizing conditions (anti-CD3/CD28 + IL-4 + anti-IFNγ) and treated with vehicle or cicaprost (2 nM, 20 nM and 200 nM) for 3 d. Flow cytometry analysis was performed after gating on live, single, CD3^+^CD4^+^, and dye-positive populations for MitoTracker and MitoSOX assays. (A) Mitochondrial mass assessed by MitoTracker mean fluorescence intensity (MFI). (B) Mitochondrial superoxide levels measured by MitoSOX MFI. (C) Mitochondrial protein expression determined by the ratio of COX-1 and SDH-A (MitoBiogenesis In-Cell ELISA). Data represent 2 independent experiments (n = 3 mice/group). *P < 0.05 by 2-way analysis of variance. OD, optical density.

To evaluate mitochondrial health and biogenesis in Th2 cells, we used MitoTracker, MitoSOX, and MitoBiogenesis assays. MitoTracker analysis revealed that cicaprost reduced signal intensity in WT cells but not in IP KO cells (Fig. 3A), indicating an IP signaling-specific decrease in mitochondrial mass and membrane potential. However, MitoSOX measurements showed that cicaprost had no effect on superoxide levels in WT and IP KO cells (Fig. 3B), excluding elevated oxidative stress as a contributing factor. Interestingly, the COX-1/SDH-A ratio, reflecting the relative abundance of mitochondrial DNA- and nuclear DNA–encoded mitochondrial proteins, was increased by cicaprost in WT cells (Fig. 3C), suggesting a shift toward enhanced mitochondrial protein expression. Together, these findings indicate that PGI_2_ reprograms mitochondrial function without inducing oxidative stress or compromising mitochondrial integrity.

### PGI_2_ signaling alters Th2 cell metabolite profiles

**Figure 4.**
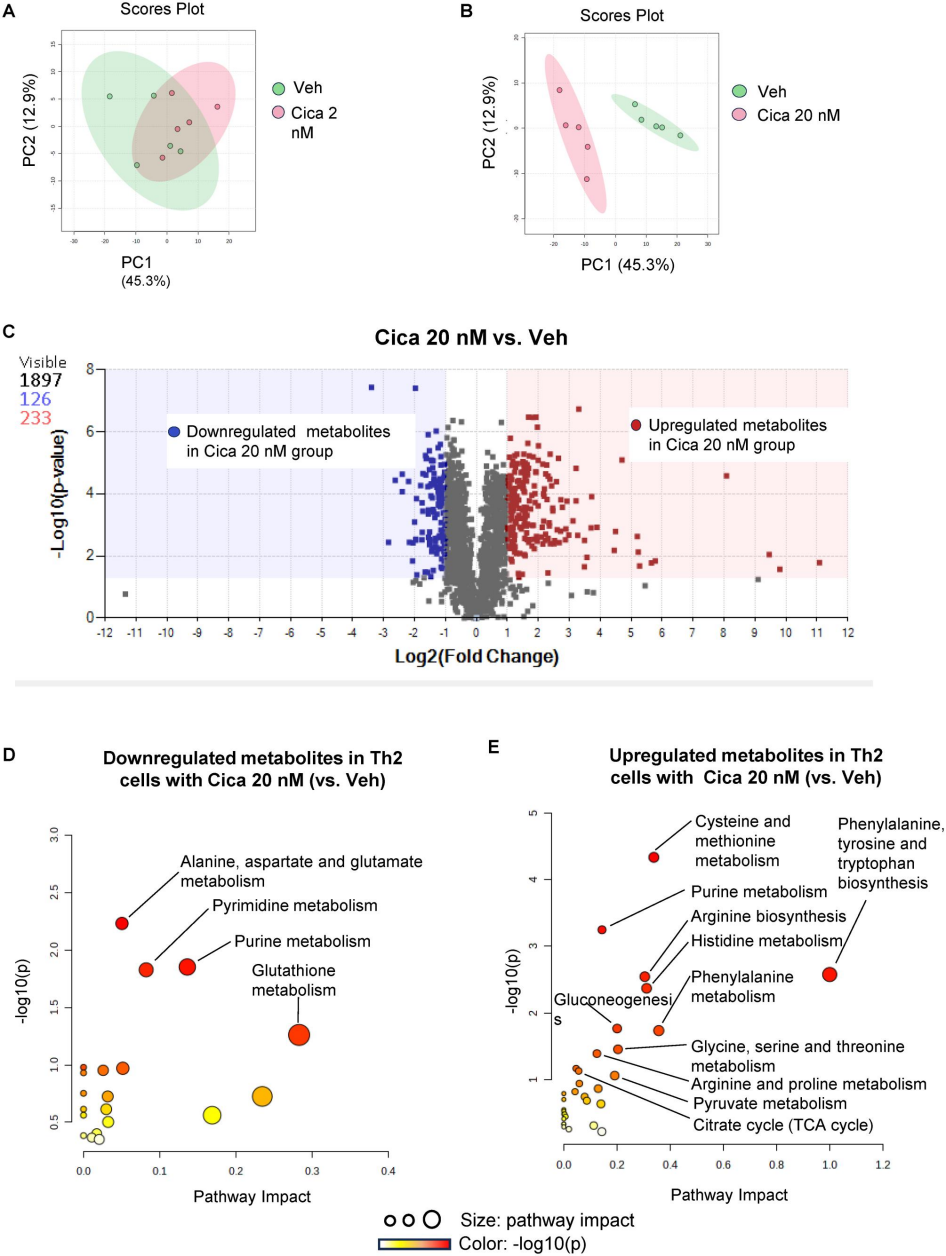
PGI_2_ signaling reprograms metabolic pathways in Th2 cells. Naïve splenic CD4^+^ T cells from WT mice were activated with anti-CD3/CD28 under Th2-polarizing conditions (IL-4 + anti-IFNγ) and treated with vehicle or cicaprost (2 nM or 20 nM) for 4 d. Metabolomic analysis revealed (A, B) principal component (PC) analysis of vehicle-treated versus cicaprost-treated samples (2 nM and 20 nM, respectively), (C) a volcano plot of differentially abundant metabolites (cicaprost 20 nM vs vehicle treatment), and (D, E) significantly altered metabolic pathways (cicaprost 20 nM vs vehicle treatment). n = 5 mice/group. TCA, tricarboxylic acid.

To explore the broader impact of PGI_2_ on Th2 cell metabolism, we performed untargeted metabolomics analyses of Th2 cells treated with cicaprost. At a lower dose (2 nM), cicaprost did not induce significant changes in metabolite profiles compared with vehicle-treated cells (Fig. 4A). However, treatment with a higher dose of cicaprost (20 nM) resulted in distinct metabolite profiles (Fig. 4B), highlighting a dose-dependent effect of PGI_2_ signaling.

A deeper analysis revealed that in cicaprost (20 nM)-treated Th2 cells, 126 metabolites were downregulated, and 233 metabolites were upregulated with changes greater than 2-fold compared with vehicle-treated cells (Fig. 4C; Table S1 and S2). These alterations indicate that PGI_2_ signaling influences a broad spectrum of metabolic processes. Metabolic pathway analysis highlighted significant changes in various pathways. Downregulated metabolites were enriched in the pathways of alanine, aspartate, glutamate, pyrimidine, purine, and glutathione metabolism (Fig. 4D), whereas upregulated metabolites were associated with pathways of cysteine, methionine, arginine, histidine, phenylalanine, glycine, and pyruvate metabolism (Fig. 4E). Upregulated metabolites were also enriched in biosynthesis pathways of arginine, phenylalanine, and glycogen (Fig. 4E). The accumulation of metabolites in multiple amino acid metabolic pathways in cicaprost (20 nM)-treated cells suggest reduced utilization of these metabolites for protein synthesis necessary for cell proliferation. The upregulation of glycogen biosynthetic pathways indicates a shift from catabolic to anabolic processes and a suppression of effector functions. These findings illustrate the broad influence of PGI_2_ signaling on Th2 cell metabolism.

### PGI_2_ signaling decreased total numbers of Arg1^+^ Th2 cells

Given the established role of arginine metabolism in T cell activation and differentiation, we investigated its regulation in our system. Cicaprost treatment reduced the total number of Arg1^+^ WT Th2 cells, which correlated directly with the decrease in total live cell numbers, without altering the mean fluorescence intensity of Arg1 (Fig. S2). The reduction in Arg1^+^ cell numbers observed in WT Th2 cells did not occur in IP KO Th2 cells, indicating that cicaprost’s suppressive effect requires IP receptor–dependent signaling (Fig. S2). These results suggest a reduction in the proportion of Arg1-expressing cells rather than a change in Arg1 expression levels per cell.

We also assessed the expression of Slc7a2/Cat2b, a high-affinity cationic amino acid transporter (for L-arginine, L-lysine, and L-ornithine) critical for T cell activation. However, Slc7a2/Cat2b messenger RNA was undetectable by RT-PCR, likely due to its low basal expression in Th2 cells under our experimental conditions.

### PGI_2_ signaling inhibits allergic lung inflammation in CD4-specific conditional IP KO mice

**Figure 5.**
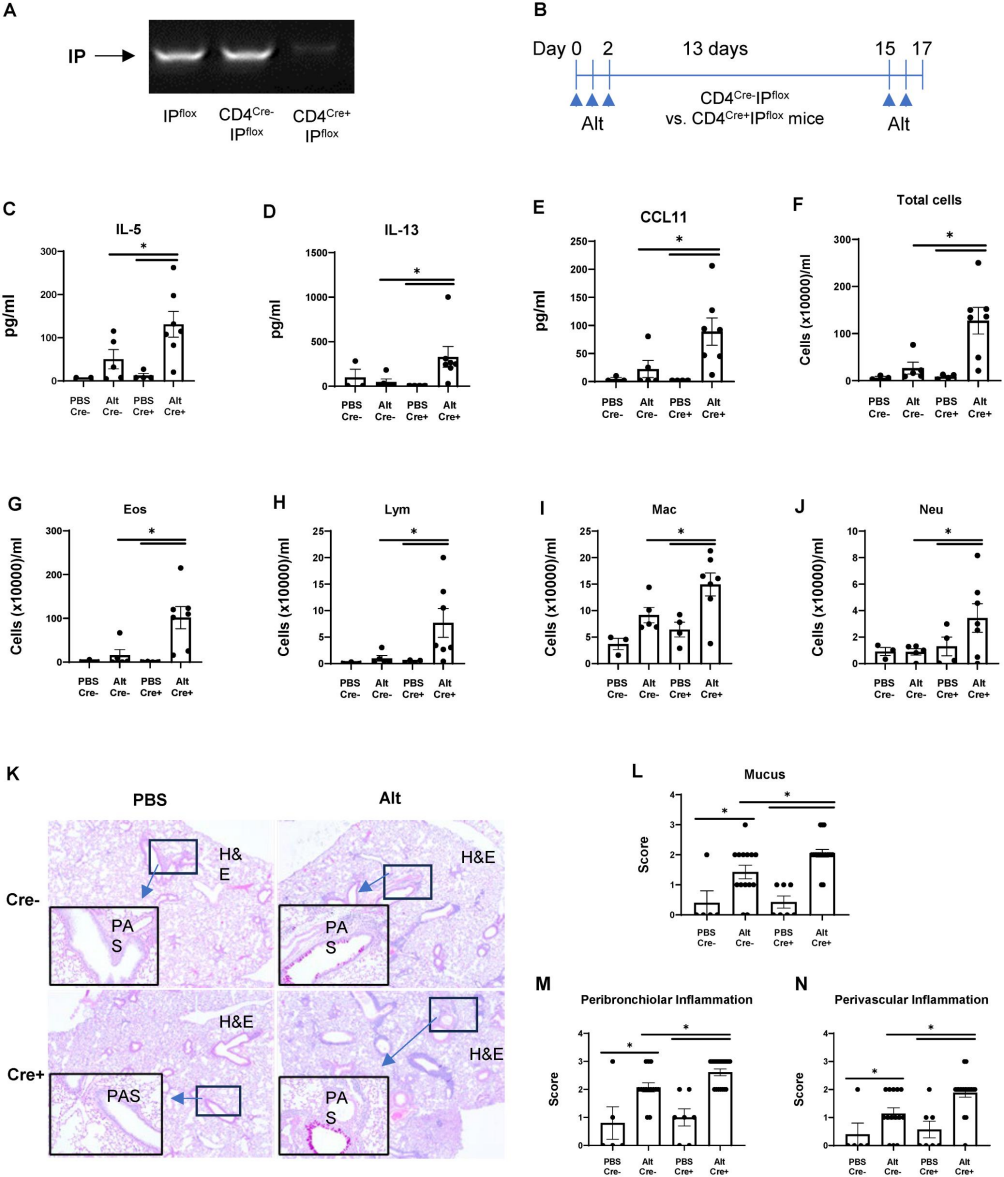
CD4^+^-specific PGI_2_ signaling attenuates *Alternaria*-induced Th2 lung inflammation. (A) IP receptor expression (RT-PCR) in CD4^+^ T cells from CD4^Cre-^IP^flox^ (control) and CD4^Cre+^IP^flox^ (IP-deficient) mice. (B) Experimental timeline: mice were intranasally sensitized/challenged daily with Alt on days 0 to 2 and 15 or 16, with mouse harvest on day 17. (C–E) IL-5, IL-13, and CCL11 levels in lung homogenates (ELISA). (F–J) BALF cellularity: total cells, eosinophils, lymphocytes, macrophages, and neutrophils. (K–L) PAS-stained mucus production (representative image and quantification). (K, M–N) Hematoxylin and eosin (H&E)–stained inflammation (representative image and scores for peribronchiolar/perivascular infiltration). Data represent 3 experiments (n = 5–18 mice/group). *P < 0.05 by 2-way analysis of variance.

To determine the physiological relevance of PGI_2_ signaling in T cells during allergic inflammation, we generated CD4^Cre+^IP^flox^ mice, which lack the IP receptor in CD4^+^ cells. RT-PCR confirmed the successful deletion of the IP receptor in CD4 T cells from CD4^Cre+^IP^flox^ mice, while CD4 T cells from CD4^Cre-^IP^flox^ mice retained IP receptor expression (Fig. 5A). We observed no compensatory upregulation of other prostaglandin receptors in IP-deficient CD4^+^ T cells (Fig. S3), consistent with our previous study in mouse lung tissue^14^ and confirming the specificity of this pathway. Using a fungal allergen (Alt)–induced allergic lung inflammation model (Fig. 5B), we observed that CD4-specific deletion of the IP receptor in CD4 T cells exacerbated pulmonary inflammation. This was evidenced by elevated levels of IL-5, IL-13, and CCL11 (Fig. 5C–E); enhanced eosinophilia; and higher numbers of total cells, lymphocytes, macrophages, and neutrophils in the BALF (Fig. 5F–J). Additionally, there was greater mucus production and more severe perivascular and peribronchiolar inflammation in CD4^Cre+^IP^flox^ mice compared with CD4^Cre-^IP^flox^ mice (Fig. 5K–N). These results indicate that the deletion of the IP receptor in CD4^+^ cells intensifies allergen-induced lung inflammation and Th2 immune responses.

## Discussion

This study highlights the critical role of PGI_2_ signaling through the IP receptor in modulating Th2 cell metabolism and immune responses. Our findings demonstrate that PGI_2_ signaling inhibits both glycolysis and mitochondrial respiration in Th2 cells, reinforcing the notion that metabolic pathways are essential for immune cell function, particularly in allergic and inflammatory diseases.^15^ These results align with recent research indicating that metabolic regulation plays a crucial role in immune cell differentiation and function.^7^^,^^8^ For instance, decreased glycolytic activity in Th2 cells inhibits their cytokine production and effector functions.^8^^,^^16^ Similarly, metabolic reprogramming of T cells has been shown to reduce their inflammatory responses, as changes in mitochondrial respiration correlate with T cell activation and survival.^3^^,^^17^ Thus, PGI_2_-mediated suppression of glycolysis and mitochondrial respiration represents a key mechanism by which Th2 cell activity is modulated.

A key discovery of this study was that PGI_2_ signaling strongly suppresses Th2 cell metabolism through IP receptor-dependent inhibition of glycolysis. This metabolic regulation correlated with reduction of GLUT1 expression, impairment of GLUT1 membrane translocation (as evidenced by decreased surface expression following cicaprost treatment), and consequent limitation of glucose uptake. This aligns with studies showing that glucose transporters like GLUT1 are critical for maintaining metabolic activation of T cells and macrophages.^18–20^ As GLUT1 serves as the primary glucose transporter in activated T cells, these effects can restrict glycolytic flux and ATP generation, ultimately decreasing Th2 cell proliferation. These findings suggest a direct mechanistic link between PGI_2_-mediated metabolic suppression and restrained Th2 cell expansion, providing new insight into how prostaglandin signaling regulates immune cell function through metabolic modulation.

To further dissect the metabolic impact of PGI_2_ signaling, we integrated Seahorse MitoStress assays with MitoTracker, MitoSOX, and MitoBiogenesis analyses to comprehensively assess the effect of cicaprost on mitochondrial function in Th2 cells. Our results demonstrate that cicaprost induced multifaceted mitochondrial dysfunction: MitoStress assays revealed that cicaprost reduced mitochondrial respiration, total ATP output, and ATP production rate, while MitoTracker data showed that cicaprost decreased mitochondrial mass and membrane potential—consistent with impaired mitochondrial integrity and biogenesis. Notably, cicaprost had no effect on superoxide levels, as shown by MitoSOX data, indicating that these functional deficits occur independently of oxidative stress. The persistence of baseline superoxide levels despite mitochondrial dysfunction suggests the engagement of redox buffering mechanisms that promote cellular survival during mitochondrial stress conditions.

Interestingly, the MitoBiogenesis assay showed cicaprost increased the COX-1/SDH-A ratio, suggesting a compensatory upregulation of mitochondrial protein expression despite overall respiratory suppression. A change in the COX-1/SDH-A ratio reflects the balance between mitochondrial- and nuclear-encoded proteins shifting, but this does not necessarily translate to a change in functional output. Changes in functional output might be negated by post-translational modifications, complex assembly dynamics, or substrate availability.

Metabolomic analysis of cicaprost-treated Th2 cells revealed significant alterations in metabolite profiles, highlighting the metabolic impact of IP signaling. Notably, we observed a dose-dependent shift in the metabolite landscape, with 126 metabolites showing downregulation and 233 metabolites exhibiting upregulation by more than 2-fold in high-dose cicaprost (20 nM)-treated Th2 cells compared with vehicle-treated controls. These changes suggest a shift from catabolic to anabolic metabolism. Specifically, downregulated metabolites were predominantly associated with key metabolic pathways such as alanine, aspartate, glutamate, pyrimidine, purine, and glutathione metabolism. These pathways are central to various cellular functions, including protein and nucleotide synthesis and redox homeostasis. Activated T cells produce reactive oxygen species, which trigger the antioxidative glutathione response necessary to buffer rising reactive oxygen species and prevent cellular damage.

Conversely, the upregulated metabolites in cicaprost-treated Th2 cells were enriched in pathways involving cysteine, methionine, arginine, histidine, phenylalanine, glycine, and pyruvate metabolism, along with arginine, phenylalanine, and glycogen biosynthesis. The increased levels of amino acid metabolites suggest reduced utilization for protein synthesis and cell proliferation. Additionally, the elevation of metabolites related to glycogen biosynthesis in cicaprost-treated Th2 cells implies a potential increase in energy storage, supporting the enhanced anabolic processes observed. This shift in glucose metabolic profiles aligns with previous reports that cicaprost suppresses cell growth and differentiation.^21^ These findings suggest that metabolic reprogramming may be integral to the cellular responses of Th2 cells induced by cicaprost. Further investigations are needed to elucidate the precise molecular mechanisms driving these metabolic changes and their functional implications in Th2 cell biology.

Our study also revealed that cicaprost treatment reduced both the total number of live Th2 cells and the population of Arg1^+^ cells. This parallel reduction suggests that PGI_2_-IP signaling may suppress Arg1 expression primarily by limiting overall Th2 cell expansion, rather than by directly inhibiting arginase-1 transcription or translation. The preserved Arg1 mean fluorescence intensity in surviving cells indicates that per-cell enzymatic capacity remains intact, potentially allowing sustained polyamine synthesis needed for cellular adaptation to metabolic stress.

Importantly, this study establishes—for the first time to our knowledge—a genetic model to dissect cell-specific functions of PGI_2_-IP signaling in vivo through the generation of CD4^Cre+^IP^flox^ mice. Our findings demonstrate that CD4^+^ T cell–intrinsic IP receptor signaling plays a nonredundant, protective role in limiting allergic pulmonary inflammation. Notably, we observed no compensatory upregulation of other prostaglandin receptors in IP-deficient CD4^+^ T cells, confirming the specificity of this pathway. When challenged with *Alternaria* allergen, mice lacking IP in CD4^+^ cells exhibited exacerbated Th2 inflammation, directly linking IP signaling in CD4^+^ cell lineage to the suppression of allergic responses. These results align with and extend our prior observations in global IP KO mice,^11^ providing cellular resolution to the mechanism by which PGI_2_ constrains pathological Th2 activation.

While the CD4^Cre^ driver also depletes IP in CD8^+^ T cells, multiple lines of evidence indicate that this population contributes minimally to allergic lung inflammation. Both our previous study using CD4^+^ or CD8^+^ T cell depletion and a study by others confirm that CD4^+^ T cells are the dominant effectors in this setting.^22^^,^^23^ Therefore, we conclude that the exacerbated pulmonary inflammation observed in CD4^Cre+^IP^flox^ mice reflects a CD4^+^ T cell–autonomous role for IP signaling. Together, these data position the PGI_2_-IP axis as a selective checkpoint for Th2-driven allergic airway inflammation, offering new insights into prostaglandin-mediated immunoregulation.

The PGI_2_-IP–mediated suppression of glycolysis and mitochondrial respiration represents a fundamental metabolic change that likely underlies its protective effects in multiple allergic airway disease models. Consistent with this, PGI_2_ analogs inhibit Th2-driven airway inflammation in both Alt and OVA models by reducing CD25 expression and IL-5/IL-13 production, as shown in prior studies,^24^ including our own.^11^ Our current work now establishes the mechanistic link between these immunoregulatory effects and metabolic reprogramming. Together, these findings demonstrate that PGI_2_-IP signaling constrains allergic inflammation through metabolic regulation of Th2 cell function. While our study focuses on Th2 cells due to their central role in allergic inflammation, PGI_2_ may similarly regulate metabolic programs in other T cell subsets implicated in allergic responses, particularly Th17 cells and T regulatory cells. Seahorse metabolic profiling of T regulatory cells revealed no significant alterations in glycolytic activity or mitochondrial respiration following cicaprost treatment (unpublished observation), suggesting Th2 subset–specific metabolic regulation. Investigating PGI_2_'s effects on Th17 cell metabolism represents a critical future direction to fully understand its immunomodulatory potential.

In summary, PGI_2_-IP signaling controls Th2 responses by reprogramming cellular metabolism, including suppression of glycolysis, mitochondrial respiration, GLUT1 expression, and mitochondrial mass and membrane potential. Our findings establish a mechanistic link between PGI_2_-mediated immunoregulation and metabolic reprogramming, reinforcing its role as a key modulator of allergic inflammation. Future comparative studies will delineate conserved versus context-dependent aspects of PGI_2_-mediated immunoregulation, further enhancing our understanding of prostaglandin biology and its therapeutic potential.

## Supplementary Material

vkaf130_Supplementary_Data

## Data Availability

All data supporting the findings of this study are available from the corresponding author upon reasonable request.
