# Supplementary material for: PGI2 signaling metabolically reprograms CD4 Th2 cells and represses allergic airway inflammation
Source: J Immunol. 2025 Jun 30;214(9):2270–80. doi: 10.1093/jimmun/vkaf130 (PMC12481032; doi:10.1093/jimmun/vkaf130)
Supplement: vkaf130_Supplementary_Data [file vkaf130_supplementary_data.pdf]

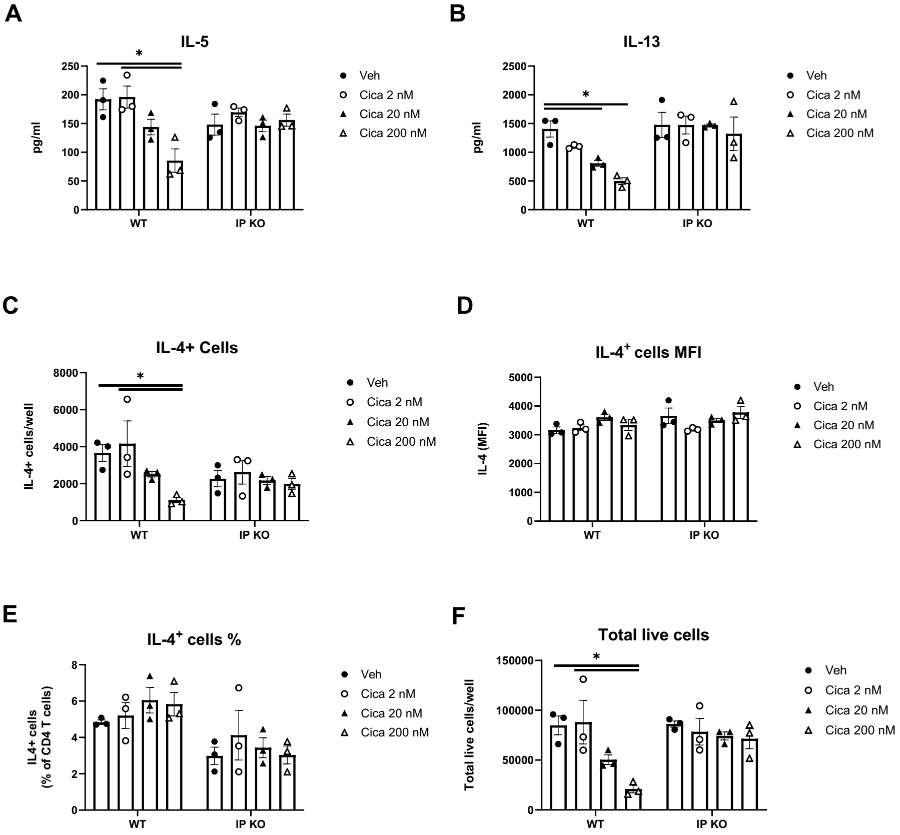

Supplementary Figure S1. PGI_2 signaling inhibits IL-5 and IL-13 production by Th2 cells. Naïve splenic $CD4^+$ T cells from WT and IP KO mice were stimulated with anti-CD3 and anti-CD28 under Th2-polarizing conditions (IL-4 + anti-IFNγ) for 3 days. Cells were treated with vehicle or cicaprost (2 nM, 20 nM, or 200 nM) at the start of culture. Levels of **(A)** IL-5 and **(B)** IL-13 in supernatants were measured by ELISA. **(C–F)** Intracellular IL-4 was assessed by flow cytometry after 5 h of PMA/ionomycin stimulation. Cells were gated for live, single, $CD3^+CD4^+$, and $IL\text{-}4^+$ populations. **(C)** Total $IL\text{-}4^+$ cell counts. **(D)** Mean fluorescence intensity (MFI) of $IL\text{-}4^+$ cells. **(E)** Percentage of $IL\text{-}4^+$ cells. **(F)** Total live cell counts. Data (A–B) are representative of two independent experiments. Statistical significance was determined by two-way ANOVA (*$p < 0.05$; n = 3 mice/group).

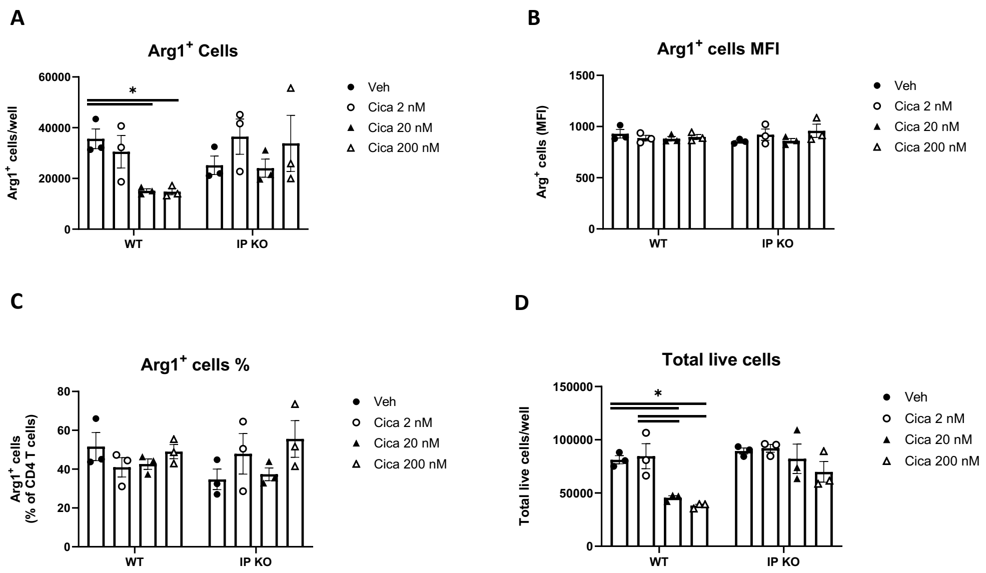

Supplementary Figure S2. PGI2 signaling reduces Arg1-expressing CD4+ T cells of WT but not IP KO mice. Naïve splenic $CD4^+$ T cells from WT and IP KO mice were polarized under Th2 conditions (anti-CD3/CD28 + IL-4 + anti-IFNγ) and treated with vehicle or cicaprost (2-200 nM) for 3 days. Cells were fixed, permeabilized, and stained for intracellular Arg1. Cells were gated for single, live, $CD4^+CD3^+$, and Arg^+ populations. **(A)** Absolute counts of $Arg1^+$ cells. **(B)** Arg1 expression levels (MFI). **(C)** Percentage of $Arg1^+$ cells. **(D)** Total viable cell counts. Data are representative of two independent experiments (n = 3 mice/group). $^*p < 0.05$ by two-way ANOVA.

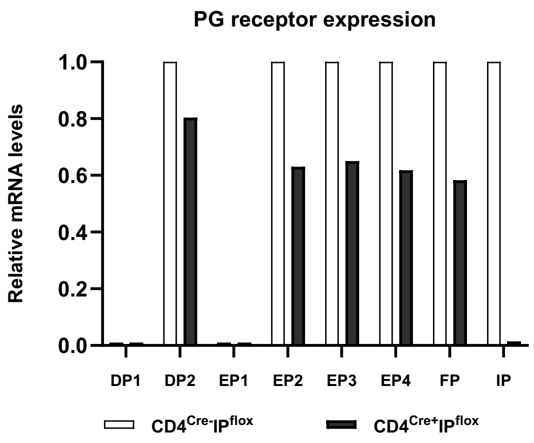

Supplementary Figure S3. IP receptor deficiency does not induce compensatory upregulation of other prostaglandin receptors. Naïve CD4+ T cells were isolated from spleens of $CD4^{Cre+}IP^{flox}$ (IP-deficient) and $CD4^{Cre-}IP^{flox}$ (control) mice. Cells from three mice per genotype were pooled for total RNA extraction, cDNA synthesis, and quantitative real-time PCR (Qiagen prostaglandin receptor primer set). mRNA expression levels of prostaglandin receptors, normalized to TATA box binding protein (Tbp) and analyzed by the ΔΔCT method. Agarose gel electrophoresis was used to confirm expected amplicon sizes for all prostaglandin receptor PCR products.

Supplementary Table S1. Downregulated metabolites (cicaprost 20 nM vs. vehicle)

1. (3-phenylpropoxy)sulfonic acid
2. 1,2,4-Trithiolane
3. 20-COOH-leukotriene E4
4. 2-Acetyl-4,5-dihydrothiazole
5. 2-Ethoxythiazole
6. 2-Octenedioic acid
7. 3-Isopropenylpentanedioic acid
8. Isocrotonic acid
9. 3-Hydroxysuberic acid
10. Gamma-Butyrolactone
11. Oxolan-3-one
12. Diacetyl
13. Ethenyl acetate
14. Methyl acrylate
15. Ethyl 2,4-dioxohexanoate
16. Diethyl fumarate
17. Diethyl L-malate
18. 3(S)-hydroxy-13-cis-docosenoyl-CoA
19. 3-Methoxymorphinan
20. Levorphanol
21. Dextrorphan
22. 3-Oxo-12,18-ursadien-28-oic acid
23. Tyromycic acid
24. 4-Fumarylacetoacetic acid
25. Maleylacetoacetic acid
26. 4-fumarylacetoacetate(2-)
27. Amifostine
28. beta-nicotinamide D-ribonucleotide
29. 1-Methylguanosine
30. 3'-O-Methylguanosine
31. Dihydroneopterin phosphate
32. Nelarabine
33. Chlorhexidine
34. Myricatomentoside I
35. Austinol
36. Icariside E5
37. Methyl 3,4,5-trimethoxycinnamate
38. Isolariciresinol 9'-O-beta-D-glucoside
39. cis-4-Decenedioic acid
40. (4-Hydroxybenzoyl)choline
41. Matsutakic acid A
42. Ilicifolinoside A
43. Citpressine I
44. Clorazepate
45. Flazine methyl ether
46. Cuminaldehyde
47. Anethole
48. 4-Phenyl-3-buten-2-ol
49. 2,3,6-Trimethylbenzaldehyde
50. Duryl aldehyde
51. 2,4,6-Trimethylbenzaldehyde
52. 2',4'-Dimethylacetophenone
53. Butylparaben
54. Zingerone
55. Isobutyl salicylate
56. Butyl salicylate
57. Methoxyeugenol
58. p-Ethylacetophenone
59. Ethyl 4-ethoxybenzoate
60. Cycasin
61. Cyclobrassinone
62. Demethylphylloquinone
63. Manglupenone
64. Ganoderal A
65. Deoxyuridine
66. Edetic Acid
67. Glutathione
68. Aristolodione
69. Hallacridone
70. Glycodiazine
71. Glycylproline
72. Prolylglycine
73. Hydroxyprolyl-Tyrosine
74. Tyrosyl-Hydroxyproline
75. Aspartame
76. N-lactoyl-Tryptophan
77. cyclic Melatonin
78. gamma-Glutamylphenylalanine
79. Phenylalanyl-Glutamate
80. Glutamylphenylalanine
81. D-Ribulose 5-phosphate
82. Xylulose 5-phosphate
83. Ribose 1-phosphate
84. D-Ribose 5-phosphate

85. Shikimic acid
86. D-Xylulose 1-phosphate
87. D-Arabinose 5-phosphate
88. 2-Isopropyl-3-oxosuccinate
89. Beta-L-arabinose 1-phosphate
90. Coumarin-4-carboxylic acid
91. 6-Formylumbelliferone
92. Dimethyl 2-oxoglutarate
93. Isoleucyl-Methionine
94. Leucyl-Methionine
95. alpha-Amylcinnamyl acetate
96. Heptyl cinnamate
97. Demethoxyshogaol
98. Methionyl-Isoleucine
99. Methionyl-Leucine
100. Alnustone
101. Latanoprost
102. Lisdexamfetamine
103. Methantheline
104. Mepenzolate
105. Mimosine
106. (R)-Dihydromaleimide
107. Mizoribine
108. N-Benzoylaspartic acid
109. N-Decanoylglycine
110. N-Lauroylglycine
111. Ethyl menthane carboxamide
112. N-Nonanoylglycine
113. N-Undecanoylglycine
114. Panaxytriol
115. Panaxacol
116. Longistylin A
117. Peperinic acid
118. Amyl 2-furoate
119. Furfuryl pentanoate
120. Isoamyl 2-furoate
121. Propyl propane thiosulfonate
122. Di-2-propenyl sulfide
123. Physalin E
124. Pyrroloquinoline quinone
125. Azosemide
126. S-Carboxymethyl-L-cysteine

Metabolomic analysis revealed significant downregulation (fold change >2, p<0.05 by ANOVA) of the listed metabolites in cicaprost (20 nM)-treated Th2 cells compared to vehicle controls.

Supplementary Table S2. Upregulated metabolites (cicaprost 20 nM vs. vehicle)

1. (-)-Epigallocatechin sulfate
2. Zeaxanthin
3. Lutein
4. Cryptoxanthin epoxide
5. Cryptoflavin
6. Aurochrome
7. Lycophyll
8. Isozeaxanthin
9. Cryptocapsin
10. Semi-beta-carotenone
11. Lactucaxanthin
12. Piperenol A triacetate
13. Doxepin N-oxide glucuronide
14. 1-Methylhistidine
15. Neryl glucoside
16. (S)-alpha-Terpinyl glucoside
17. D-Linalool 3-glucoside
18. Perilloside C
19. Menthol-glucoronide
20. 1-Methylinosine
21. Arabinopyranobiose
22. Xylobiose
23. Arabinofuranobiose
24. Pteroside P
25. Tetrahydrocurcumin
26. 4,5-Dihydroniveusin A
27. Licoriphenone
28. Lirioresinol A
29. 6',7'-Dihydroxybergamottin
30. Hydroxymyricanone
31. 2-Amino-3-oxoadipate
32. D-N-(Carboxyacetyl)alanine
33. FAPy-adenine
34. N-Formyl-L-glutamic acid
35. N-Acetyl-L-aspartic acid
36. 2-Ethyl-5-methylthiophene
37. 2-Propylthiophene
38. 2-Hydroxyethanesulfonate
39. Ethyl hydrogen sulfate
40. Sulfacetamide
41. 2-Methoxyacetaminophen sulfate
42. 2-Phosphoglyceric acid
43. Acetylphosphate
44. 2-Phospho-D-glyceric acid
45. Phosphonoacetate
46. 2-phosphonato-D-glycerate(3-)
47. 3-Furanmethanol glucoside
48. O-Demethylfonsecin
49. 3-Galactosyllactose
50. 4-beta-Laminaribiosylglucose
51. Neokestose
52. 6-Kestose
53. Fagopyritol B2
54. 3-beta-Gentiobiosylglucose
55. Nephritogenoside
56. Maltotriose
57. Raffinose
58. Dextrin
59. Gentiotriose
60. Galactotriose
61. Panose
62. Gentianose
63. Fagopyritol A2
64. Melezitose
65. Levan
66. 6-O-Glucosylmaltose
67. Umbelliferose
68. Sophorotriose
69. 1-Kestose
70. 3'-Hydroxy-T2 Toxin
71. 3-O-Feruloylquinic acid
72. Berberrubine
73. 4-(Nitrosoamino)-1-(3-pyridinyl)-1-butanone
74. Sclareapinone
75. 8-Methoxygravelliferone
76. Sagequinone methide A
77. 5-Geranyloxy-7-methoxycoumarin
78. Guaiacin
79. (all-E)-Crocetin
80. Macelignan
81. Verimol H
82. Lactosamine
83. Alanyl-Proline
84. Prolyl-Alanine

85. Serylvaline
86. Valyl-Serine
87. N6-Acetyl-5S-hydroxy-L-lysine
88. Arabinonic acid
89. Ribonic acid
90. N,N'-dinitrosopiperazine
91. 2,3,4,5-Tetrahydroxypentanoic acid
92. L-Lyxonate
93. L-Xylonate
94. Arginyl-Proline
95. Prolyl-Arginine
96. N-(1-Deoxy-1-fructosyl)leucine
97. Bisnorbiotin
98. 6-Aminopenicillanic acid
99. Capillin
100. Chloromarmin
101. Atovaquone
102. D-Glucuronic acid
103. Galacturonic acid
104. Iduronic acid
105. Pectic acid
106. Pectin
107. 3-Dehydro-L-gulonate
108. 5-Keto-D-gluconate
109. 2-Keto-L-gluconate
110. L-Altruronic acid
111. Aldehydo-L-iduronate
112. Cisapride
113. Cotinine glucuronide
114. Cysteineglutathione disulfide
115. Glyceryl lactopalmitate
116. Cysteinyl-Cysteine
117. 3-(4-Hydroxybenzoyl)epicatechin
118. De-O-methylsimmondsin
119. Deoxycytidine
120. Glycogen
121. Maltotetraose
122. Stachyose
123. Mannan
124. Fagopyritol B3
125. Bifurcose
126. Neobifurcose
127. Sesamose
128. Citbismine C
129. Glycylproline
130. Prolylglycine
131. Hydroxyethylpromethazine
132. Asparaginyl-Hydroxyproline
133. Hydroxyprolyl-Asparagine
134. 2-hydroxyacrylic Acid
135. Ascorbic acid
136. Malonic semialdehyde
137. Glucosereductone
138. Pyruvic acid
139. D-Glucurono-6,3-lactone
140. Fructose 6-phosphate
141. Myo-inositol 1-phosphate
142. Galactose 1-phosphate
143. Dolichyl phosphate D-mannose
144. Fructose 1-phosphate
145. Mannose 6-phosphate
146. Inositol cyclic phosphate
147. D-Myo-inositol 4-phosphate
148. Glucose 6-phosphate
149. Glucose 1-phosphate
150. Inositol phosphate
151. Beta-D-Glucose 6-phosphate
152. D-Tagatose 1-phosphate
153. D-Mannose 1-phosphate
154. Sorbose 1-phosphate
155. Beta-D-Fructose 2-phosphate
156. 1D-myo-Inositol 3-phosphate
157. D-Tagatose 6-phosphate
158. Dracunculin
159. Buntansin A
160. D-fructose 1-phosphate
161. L-Acetylcarnitine
162. Tranexamic Acid
163. (S)-Homostachydrine
164. N-lactoyl-Leucine
165. Aminoadipic acid
166. Acetylhomoserine
167. Glutamate, gamma-methyl ester
168. N-Acetylthreonine
169. N-methyl-L-glutamic Acid
170. hydroxybutyrylglycine
171. DL-Homocystine
172. L-Homocystine

173.beta-Arabinose
174.D-Ribose
175.Arabinofuranose
176.D-Apiose
177.L-Ribulose
178.Galactonic acid
179.Gluconic acid
180.Gulonic acid
181.D-Xylose
182.L-Arabinose
183.Aldehydo-D-xylose
184.2-Deoxyribonic acid
185.D-Ribulose
186.L-Threo-2-pentulose
187.D-Xylulose
188.D-Arabinose
189.2-Deoxypentonic acid
190.cis-Aconitic acid
191.trans-Aconitic acid
192.D-Glucaro-1,4-lactone
193.Citric acid
194.Isocitric acid
195.D-threo-Isocitric acid
196.2,3-diketogulonate
197.Xanthine
198.Oxypurinol
199.6,8-Dihydroxypurine
200.4-Hydroxybenzoic acid
201.Salicylic acid
202.3-Hydroxybenzoic acid
203.Coriandrin
204.Sesamol
205.Wyeronic acid
206.phenyl 2,3-dihydroxybenzoate
207.Indolelactic acid
208.N-Acetyl-L-tyrosine
209.Indoleacetaldehyde
210.5-Methoxyindoleacetate
211.Cinnamoylglycine
212.Edulitine
213.3-Indolehydracrylic acid
214.N-Hydroxy-1-aminonaphthalene
215.2-hydroxyphenylpropionylglycine
216.Isoniazid
217.Isovalerylglutamic acid
218.Suberylglycine
219.N-(1-Deoxy-1-fructosyl)proline
220.Lamivudine sulfoxide
221.L-Cystine
222.L-Glutamic acid 5-phosphate
223.L-Histidine
224.L-2-Amino-3-(1-pyrazolyl)propanoic acid
225.2-Oxoarginine
226.N-(1-Deoxy-1-fructosyl)glycine
227.Fructoseglycine
228.N-Acetyl-L-glutamate 5-semialdehyde
229.N-(1-Deoxy-1-fructosyl)tyrosine
230.N-(1-Deoxy-1-fructosyl)valine
231.Phosphoenolpyruvic acid
232.S-aminomethyldihydrolipoamide
233.S-Carboxymethyl-L-cysteine

Metabolomic analysis revealed significant upregulation (fold change >2, $p<0.05$ by ANOVA) of the listed metabolites in cicaprost (20 nM)-treated Th2 cells compared to vehicle controls.
